# Offcut Substrate-Induced Defect Trapping at Step Edges

**DOI:** 10.1021/acs.nanolett.4c00832

**Published:** 2024-04-26

**Authors:** Nicolas Bonmassar, Georg Christiani, Gennady Logvenov, Y. Eren Suyolcu, Peter A. van Aken

**Affiliations:** Max Planck Institute for Solid State Research, Heisenbergstraße 1, 70569 Stuttgart, Germany

**Keywords:** Defect engineering, Bidirectional growth, Step edge interface, Oxide molecular beam epitaxy, Scanning transmission electron microscopy

## Abstract

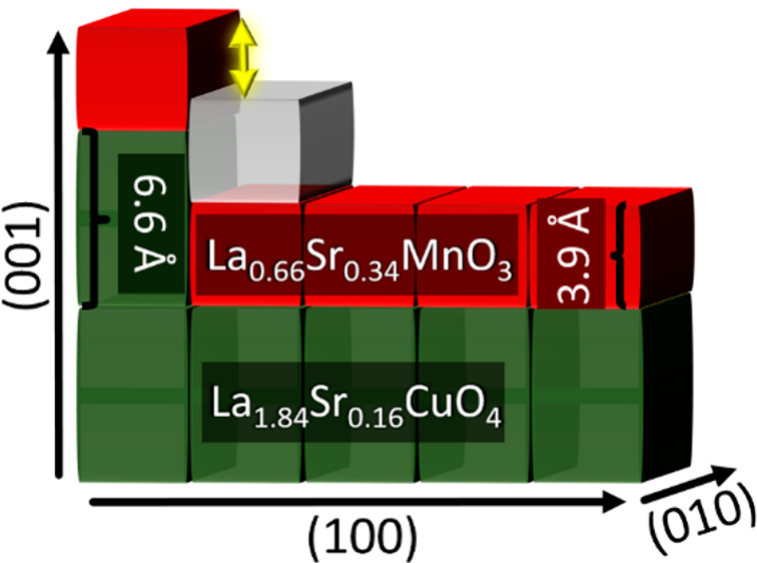

We report step edge-induced localized defects suppressing subsequent antiphase boundary formation in the bulk structure of a trilayer oxide heterostructure. The heterostructure encompasses a layer of La_0.66_Sr_0.34_MnO_3_ sandwiched between a superconducting La_1.84_Sr_0.16_CuO_4_ bottom layer and an insulating La_2_CuO_4_ top layer. The combination of a minor *a*-axis mismatch (0.11 Å) and a pronounced *c*-axis mismatch (2.73 Å) at the step edges leads to the emergence of localized defects exclusively forming at the step edge. Employing atomically resolved electron energy-loss spectroscopy maps, we discern the electronic state of those structures in the second La_0.66_Sr_0.34_MnO_3_ unit cell near the step edge. In particular, a reduction in the pre-edge region of the O-*K* edge indicates the formation of oxygen vacancies induced by the strained step edge. This study underscores our capability to control defects at the nanoscale.

Precise control of defect formation is of paramount importance for spintronics and electronic devices, as these defects can strongly affect material conductivity,^1,2^ magnetism,^3^ and ultimately device functionality.^4^ The manipulation of defects at the step edges, which can induce antiphase boundaries (APBs) plays an important role in catalysis,^5^ where their presence can drastically influence surface interactions,^6^ reaction pathways,^7^ and catalytic efficiency.^6^ Hence, understanding the underlying mechanism governing the formation and control of defects presents transformative prospects for elevating both device performances and catalytic processes.^8,9^ Recent investigations have unveiled the formation of APBs, notably originating at the step edges of substrate terraces.^10^ Furthermore, it has been demonstrated that two APBs tend to merge when neighboring steps closely align to minimize their surface energy.^10^ We used specific offcut substrates, ensuring uniform terrace widths, and consequently reducing the distances between step edges resulting in a high density of step edges.^11^ Ozone-assisted molecular beam epitaxy (MBE) guided by reflection high-energy electron diffraction (RHEED) stands as the forefront technique, ensuring high quality complex oxide heterostructures.^12,13^ This approach not only achieves exceptional film quality but also empowers precise control over the growth of individual unit cells.^14,15^ Complex oxides have established themselves as record holders across an array of properties, including high-*T*_*C*_ superconductivity,^16−18^ colossal magnetoresistance,^19^ and multiferroicity,^20^ underscoring their exceptional versatility and performance.^21^

Here, we focus on the profound impact of large offcut angles, yielding nanometer-scale terrace widths of a mere five to seven unit cells. This structural design manifests in the formation of localized defects at step edges. To probe this phenomenon, we employed scanning transmission electron microscopy with electron energy-loss spectroscopy (STEM-EELS) and high-angle annular dark-field (HAADF) imaging. We complement these advanced imaging techniques with extensive data analysis methodologies such as energy-loss near-edge structure analysis (ELNES) and geometric phase analysis (GPA), to examine the formation of defects.^22^ Through this multifaceted approach, we unveiled the formation of defects at the step edge but also discerned their electronic- and crystallographic characteristics, leading to the suppression of APBs.

Initially, we focus on a trilayer consisting of a La_0.66_Sr_0.34_MnO_3_ (LSMO) layer with a perovskite-type pseudocubic 113 crystal structure sandwiched between a superconducting La_1.84_Sr_0.16_CuO_4_ (LSCO) bottom layer with tetragonal K_2_NiF_4_ type (or 214) crystal structure and an insulating La_2_CuO_4_ (LCO) top layer. The trilayer was grown on (001) oriented LaSrAlO_4_ (LSAO) with an offcut angle of 15° toward the *a*-axis, allowing for the study of defect formation at step edges occurring every 1–2 nm. On top of that, the use of high-angle offcut substrates allows for the bidirectional growth of a heterostructure, where ferromagnetic layers (LSMO) are in the same plane as the superconducting (LSCO) or the antiferromagnetic (LCO) layers.^23^

A minor *a*-axis mismatch of 0.11 Å and a substantial *c*-axis mismatch of 2.73 Å arise at the step edge. Note that for our calculations, we used the bulk values from literature for a tetragonal half-unit cell LSCO and a pseudocubic approximation for LSMO.^24,25^Figure 1a illustrates the dislocations at the step edges (indicated by dashed yellow boxes), a direct outcome of the pronounced *c*-axis mismatch. The green lines correspond to the widths of the initial substrate terraces, marking the origin of the subsequent heterostructure. Focusing on the LSAO/LSCO interface, no defects are detected. Figure 1b depicts the formation of the defective unit cell. The two unit cells of LSMO (red and blue spheres) sum up to a total out-of-plane value of 7.76 Å, whereas a LSCO half-unit cell (red and green spheres) has a *c*-axis value of 6.61 Å. The effect of this mismatch is pointed out by the yellow arrow. This concept is proven by atomically resolved EELS elemental mapping (Mn: blue, La: red, and Cu: green) showcased in Figure 1c. Here, the defective unit cell can be identified as the second LSMO unit cell in proximity to the step edge, highlighted by the dashed yellow rectangle. Lattice adaptation to the structural mismatch and chemical intermixing of the Cu atoms from LSCO into the second LSMO unit cell at the step edge leads to this localized defect formation, while the variation of the LSMO layers’ thickness can be attributed to step bunching due to imperfect substrates and step bunching occurring during growth. Cation distribution profiles and maps for individual elements are shown in SI Figure 1.

**Figure 1:**
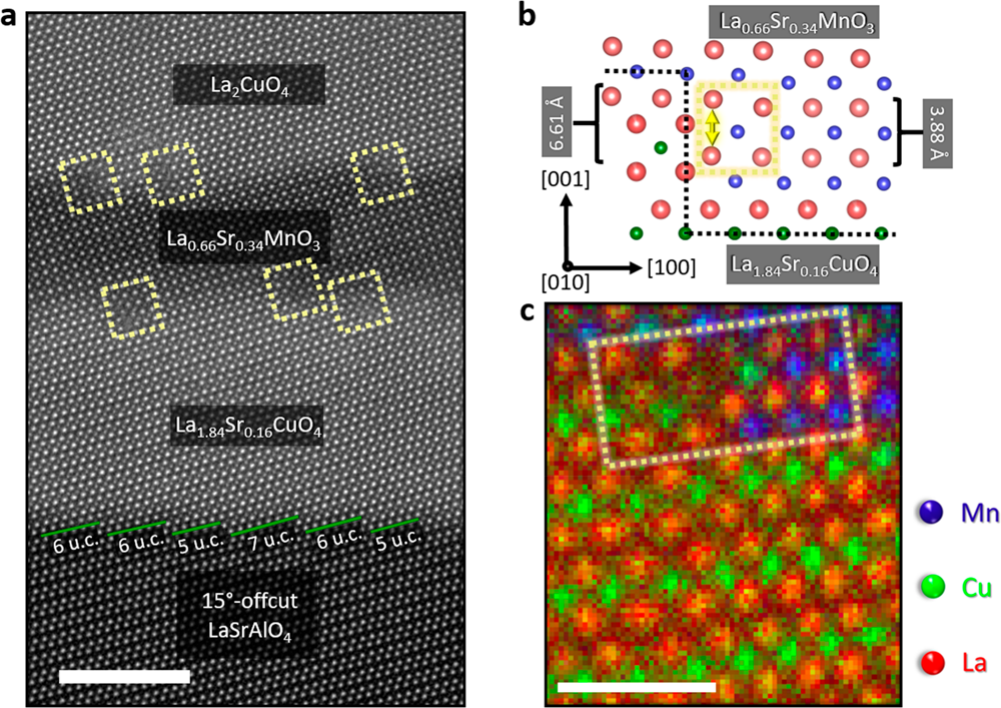
**Step edge-induced defect formation.** (**a**) Overview HAADF image of the trilayer system, LSCO-LSMO -LCO, grown on LSAO substrate possessing a 15° offcut angle toward the *a*-axis. The dashed yellow boxes highlight the defective unit cell at the step edges, while the variable terrace widths of the substrate are shown by the tracing green lines. The scale bar is 5 nm. (**b**) Conceptual representation of the mechanism underpinning the formation of defects at the step edge. Due to the considerable *c*-axis mismatch between LSMO and LSCO, the system forms a defect, indicated by the yellow square.^26,27^ The black dashed line highlights the step edge interface between LSCO and LSMO. (**c**) Atomically resolved EELS mapping shows that the defect is formed in the second LSMO unit cell. The dashed yellow rectangle highlights the step edge region. The white scale bar corresponds to 1 nm.

Moreover, the mismatch between the individual layers results in a complicated structure at the step edge as indicated by the yellow dashed box in Figure 1c. Here, the Cu B-site cation (green) within the LSCO phase undergoes a positional exchange with the La A-site cation (red) of the LSMO phase. Simultaneously, the La A-site cation (red) within the LSCO phase executes a positional exchange with the Mn B-site cation (blue). Despite these local irregularities, the superconducting behavior of LSCO is confirmed by mutual inductance response presented in SI Figure 2, and the *in situ* RHEED images obtained during growth (SI Figure 3) evidence the absence of any defect formation. In addition, there is no APB formation in the subsequent LSMO or LCO layer, which are typically found at step edges with large lattice mismatches.^10^

**Figure 2:**
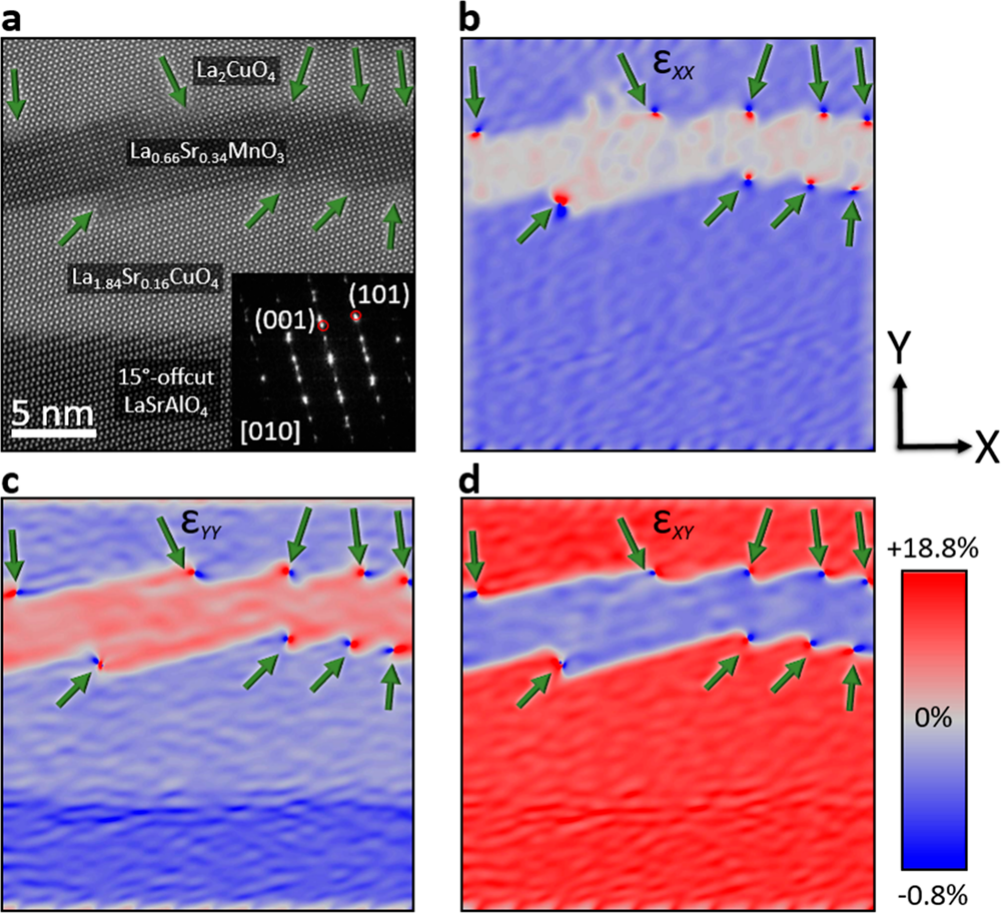
**Strain-induced lattice deformation in highly perturbed materials.** (**a**) HAADF image highlighting the specific area used for the strain calculation. The inset shows the FFT, revealing reciprocal lattice vectors g_1_ (001) and g_2_ (101) denoted within red circles, which have been used for the strain analysis. The used g-vectors can be assigned to the pseudocubic approximation of LSMO. (**b**) Lattice mismatch in the *X*-direction of the image. (**c**) Distinctive deformations in the *Y*-direction of the image. (**d**) Shear strain highlights the mean lattice dilatation. The green arrows point out step edges.

**Figure 3:**
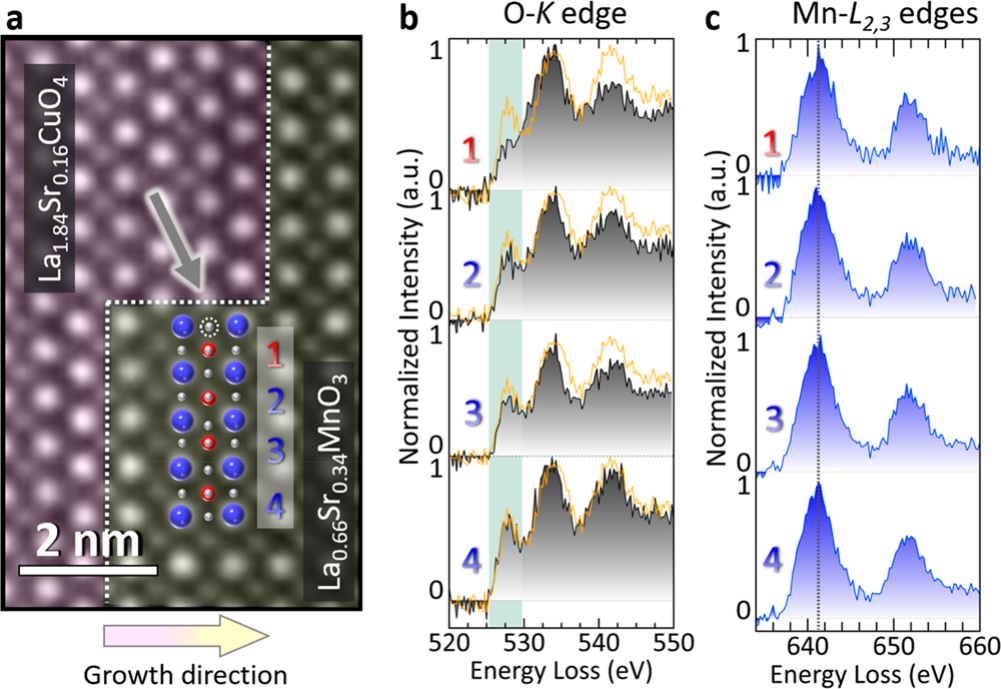
**Insight into the electronic structure at the step edge.** (**a**) HAADF image highlighting a step edge at the LSCO-LSMO interface with a pink and a yellow background, respectively, delineated by dashed white lines. The blue numbers annotate the Mn atoms beyond the deformed unit cell, while the red number denotes the precise location of the LSMO unit cells, in direct proximity to the step edge. (**b)** Atomically resolved ELNES of the four LSMO unit cells, finely dissecting the pre-edge region (green background) of the O-*K* edge. The orange profile was obtained from the middle part of the LSMO thin film and acts as a reference for the step edge region. (**c**) Similar analysis utilizing the Mn-*L*_2,3_ edges for the four LSMO unit cells.

Figure 2 presents discernible strain variations within the heterostructure, utilizing geometric phase analysis. Conventionally, strain mappings have been applied to materials exhibiting subtle lattice distortions. However, in the context of this investigation, significant *c*-axis mismatches of up to 18% introduce complexities that may challenge straightforward interpretation of the data. Our focus is exclusively on the step edge regions, where a correlation between large lattice mismatches and localized strain effects is deduced from the strain mapping. Figure 2a shows an overview HAADF image presenting the region of interest for the strain analysis. The inset in Figure 2a corresponds to a fast Fourier transformation of the HAADF image, highlighting the presence of reciprocal lattice vectors g_1_ (001) and g_2_ (101), encircled in red. Both g-vectors correspond to the sandwiched pseudocubic LSMO layer and are carefully selected to enhance the visibility of the strain states at the step edge interfaces. The LSMO layer was used as a reference area and the spatial resolution of the strain mapping is 1.15 nm. In Figure 2b, the strain in the *X*-direction is shown, revealing strongly strained materials at the LSCO/LSMO and LSMO/LCO step edges. Figure 2c presents the strain in the Y direction, highlighting the significant strain localization at the step edge region indicated by the green arrows. The step edge region, denoted by the green arrows, emerges as a hotspot of tensile strain that can introduce oxygen vacancies.^28,29^ Shear strain distributions (Figure 2d), unraveling tensile strain (red) within the LSAO, LSCO, and LCO layers, as indicated by the green arrows, ascribed to the relatively smaller a- and much higher c-axes of LSAO, LSCO, and LCO compared to the reference area LSMO. Moreover, the observed shear strain further reinforces the correlation between localized structural perturbations and the distinct oxidation states of Mn atoms within the step edge regions. While tensile strain can drive the formation of oxygen vacancies,^30,31^ we exercise caution in attributing their formation at the step edge solely based on strain analysis. Therefore, our efforts are shifting toward atomically resolved ELNES analyses, to provide a conclusive understanding of the step edge region.

Figure 3a displays an HAADF image showcasing the step edge, with distinct regions marked by four unit cells of LSMO and the interface between the LSCO (pink shade) and the LSMO (yellow shade) through a white dashed line. Note that the dashed white lines go through the bottom and top B-site cations of the LSCO and LSMO unit cells, respectively, to highlight the formation of intermixed Cu–Mn materials. This type of intermixing is described elsewhere.^32^ The gray arrow and the dashed circle point out the position of the region of interest at the step edge. We focus on the pre-edge region of the O-*K* edge, recognized for its accessibility in observing and analyzing electronic transformations arising from the hybridization of oxygen 2p orbitals with transition metal 3d t_2g_ and e_g_ orbitals. This approach allowed us to gain valuable insights into the electronic states at the atomic scale despite the inherent limitations of energy resolution in atomically resolved ELNES depicted in Figure 3b and 3c. Within this pre-edge region, there are discernible changes characterized by a significant decrease in the intensity of the prepeak feature, highlighted against a green background. A 2D mapping of the O–K prepeak of different regions is shown in SI Figure 4, where a decrease in the O-*K* pre-edge is directly visualized in the step edge region. This reduction in intensity can correspond to a reduction in the Mn valence state.^33^ This finding concurs with established reports, where the pronounced tensile strain at the step edge, arising from the substantial *c*-axis disparity between LSCO and the smaller *c*-axis of LSMO, can increase the oxygen vacancy concentration and, therefore, introduce electrons into the system, subsequently reducing the Mn valence.^29,34^ Another explanation in the subtle changes in the prepeak region of the O-*K* edge at the step edge, could be chemical intermixing between Cu and Mn cations as indicated in Figure 1c. Yet, the complexity of this scenario amplifies given the role of the LSCO layer, acting as an in-plane compressive force upon the LSMO layer. This interplay of tensile and compressive strains at the step edge gives rise to a characteristic electronic structure, distinct from either strain in isolation. Therefore, we compared the pre-edge region of the center of our LSMO layer (orange profile) to directly compare each of the four assigned unit cells with layers farther away from the interfaces to LSCO and LCO. Our results show that the first three unit cells in direct proximity to the step edge have reduced prepeaks, whereas the fourth unit cell shows a prepeak of a similar height to the center part of our LSMO layer, which is in agreement with the locally strained step edge region shown in Figure 2. In addition, the residual changes regarding the peaks at higher energy losses could be due to the La/Sr ratio differences in this region, which can alter the electronic fine structure of the O-*K* edge.^35^ The effect of strain on the physical properties originating from the substrate has been reported and discussed elsewhere.^32^ Note that, within the experimental energy resolution and signal-to-noise ratio in our atomically resolved EELS measurements, we can not resolve the subtle peak shifts in the Mn-*L*_3_ edge, c.f. Figure 3c. As a result, these nuanced shifts in the edge could not be accurately resolved or characterized in terms of an *L*_3_*/L*_2_ ratio analysis, which needs a sufficient signal-to-noise ratio. However, our fine structure analyses in the prepeak of the O-*K* edge indicate either the reduced oxidation states of the Mn atoms at the step edge via the formation of oxygen vacancies, or the mixing of Cu and Mn atoms leading to distorted unit cells, consistent with the pronounced lattice distortions observed in our strain maps in Figure 2b, **c**. This holds true for all step edges observed in our study, c.f. SI Figure 4.

**Figure 4:**
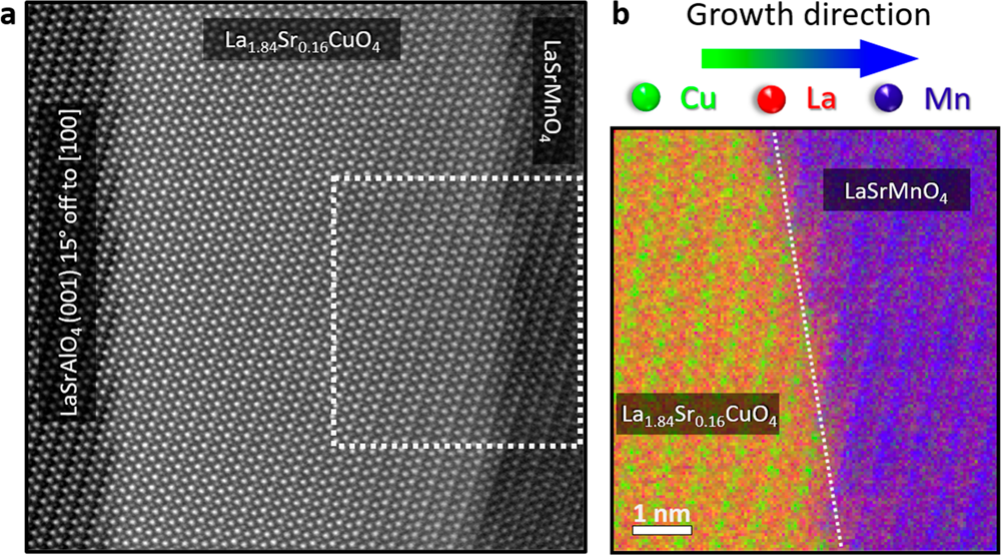
**Unveiling coherent interfaces with structurally congruent materials.** (**a**) HAADF overview image delineating the bilayer structure. The first interface is the LSAO substrate (evidenced by dark contrast on the left side) next to the LSCO (bright contrast in the middle), positioned in the middle of the image. The second LSCO-LaSrMnO_4_ interface (dark contrast on the right-hand side) is depicted on the right-hand side. The dashed white square showcases the area for the EELS elemental mapping. (**b**) Atomically resolved EELS elemental mapping with La in red, Cu in green, and Mn in blue shows the high chemical quality of the bilayer system. White line highlights the interface between the cuprate and the Manganite layer.

In materials characterized by minimal lattice mismatches, localized step edge defects are absent, as indicated in Figure 4. Particularly, the examination of LSCO and LaSrMnO_4_ bilayers with tetragonal 214 crystal structure grown on an (001) oriented LSAO substrate with a 15° offcut toward the *a*-axis, distinguished by a mere 2 pm difference in *c*-axis values,^36^ reveals an absence of detected defects, underscoring the role of lattice coherence in influencing the formation of defects at the step edge. Utilizing a 15° offcut angle for the LSAO substrate ensures identical conditions to those governing the LSCO-LSMO-LCO trilayer within our experimental setup. The absence of defect formations is further demonstrated by atomically resolved EELS elemental mappings, shown in Figure 4b. The individual elemental maps are shown in SI Figure 5.

Utilizing the geometric attributes of precise offcut substrates as a strategic tool coupled with the unique advantages offered by layer-by-layer growth through oxide MBE, we present a compelling demonstration of tunable step edge defects at the subnanometer scale. The use of offcut substrates leads to trapping of defects at the step edges. We demonstrated that these step edge-induced defects are localized and do not penetrate the bulk of the subsequent thin film. Furthermore, in contrast to studies on non- or low-offcut substrates using similar materials,^10^ the formation of APBs is suppressed, when growing on high-offcut angle substrates. This suppression is due to the higher surface energy resulting from the larger number of open bonds at the step edges compared to low-angle offcut substrates, which have fewer step edges. We attribute this defect trapping to the pronounced *c*-axis mismatch between the bottom and top epitaxial layers. The geometry-assisted formation of trapped defects induces structural rearrangements at the subnanometer scale, consequently introducing novel chemical and electronic properties in these localized regions. These controllable local inhomogeneities thus stand as a promising tool for fine-tuning the electronic properties of oxide materials. In addition, defect-free step edges, c.f. Figure 4, due to the structural match between LSCO and LaSrMnO_4_ highlight the potential for future in-plane Josephson junctions. This potential arises from the notably greater in-plane coherence length of Cooper pairs in oxide high-*T*_*C*_ materials, approximating 2.4 nm, significantly surpassing the corresponding out-of-plane coherence length, approximating 0.3 nm.^18^

Our results indicate another opportunity to explore new research domains, encompassing the stabilization of high-pressure phases at step edges and the formation of cation diffusion channels. Adopting offcut substrates to thin film technology will pave the way for novel in-plane device possibilities in spintronics and electronics. Notably, the control over APBs holds a profound role in catalysis, given their potential to significantly influence surface reactivity, adsorption, and overall catalytic performance.^5,6^ The formation of localized defects and the prevention of APBs in the subsequent thin film layer benefit fundamental science and practical applications that may underpin critical technological advancements.

## RHEED-Assisted MBE Growth in Ozone

LSCO-LSMO-LSCO heterostructures were epitaxially grown on LSAO (001) (CrysTec GmbH) with a 15° offcut angle toward the (010) direction, respectively, using molecular beam epitaxy (MBE) under a highly oxidative atmosphere composed of ozone, molecular oxygen, and radical oxygen (DCA Instruments Oy). The growth process for all depositions took place at approximately 1*10^−5^ Torr and temperatures ranging from 680 to 700 °C (as measured by a pyrometer). *In situ* reflection high-energy electron diffraction (RHEED) was employed to monitor each deposited layer during the growth.

## Diamagnetic Response Signal

Measurements of mutual inductance (MI) were carried out using a two-coil configuration (parallel geometry) with an alternating current of 50 μA and a frequency of 1000 Hz to determine the real and imaginary components of the magnetic susceptibility.

## Scanning Transmission Electron Microscopy (STEM)

All specimens were thinned to electron transparency using tripod-wedge polishing, followed by ion polishing. Ar^+^ ion thinning the samples to less than 30 nm thickness was accomplished using a precision ion polishing system (PIPS II, Model 695) equipped with a cooling stage filled with liquid nitrogen to minimize sample preparation induced artifacts.STEM analyses were performed using a JEOL JEM-ARM200F transmission electron microscope equipped with a cold-field emission gun, a probe C_S_-corrector (DCOR, CEOS GmbH), and a Gatan GIF Quantum ERS electron energy-loss spectrometer. A convergence semiangle of 22 mrad, resulting in a probe size of 0.8 Å, was used for all STEM and EELS analyses. HAADF imaging was performed within a collection-angle range of 87–209 mrad. EELS data were acquired with a collection semiangle of 87 mrad. A direct electron detection camera (Gatan K2) with a 0.5 eV/channel energy dispersion (yielding an energy resolution of less than 1 eV) and a pixel dwell time of 3.7 ms were employed for all EELS experiments. For the ELNES analyses in Figure 3, a 0.25 eV/channel dispersion was used, leading to a final energy resolution of ∼0.5 eV. To reduce noise, false color-coded RGB maps were generated using principal component analysis (PCA) with 10 components. Analyses of Mn-*L*_2,3_ and O-*K* edges were carried out using raw data. Strain analysis was conducted using Geometrical Phase Analysis (GPA),^22^ a technique pioneered by Martin Hÿtch, through a commercial GPA plugin (HREM Research Inc.) integrated into Gatan DigitalMicrograph software.

## Supporting Information Available

EELS elemental mappings, Mutual inductance vs temperature measurement, *in situ* reflection high-energy diffraction pattern (PDF)
