# Supplementary material for: Offcut Substrate-Induced Defect Trapping at Step Edges
Source: Nano Lett. 2024 Apr 26;24(18):5556–61. doi: 10.1021/acs.nanolett.4c00832 (PMC11082922; doi:10.1021/acs.nanolett.4c00832)
Supplement: Supplementary file 1 — nl4c00832_si_001.pdf [file nl4c00832_si_001.pdf]

Supplementary Information: Offcut Substrate-Induced Defect Trapping at Step Edges

Nicolas Bonmassar, Georg Christiani, Gennady Logvenov, Y. Eren Suyolcu*, and Peter A. van Aken*

Max Planck Institute for Solid State Research, Heisenbergstraße 1, 70569 Stuttgart, Germany

Email: n.bonmassar@fkf.mpg.de and eren.suyolcu@fkf.mpg.de

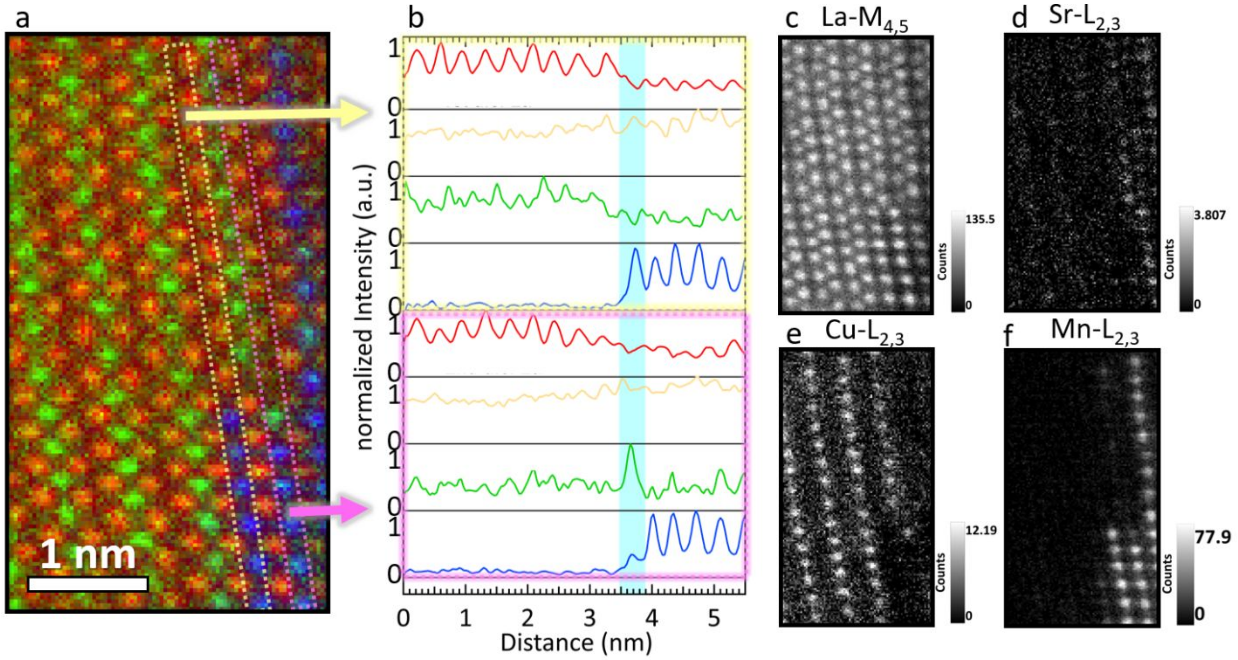

SI Figure 1: (a) EELS maps showing the elemental distribution of La (red), Sr (yellow), Cu (green), and Mn (blue). (**b**) The related elemental profiles obtained from the yellow and pink dashed rectangles are displayed. The turquoise background in panel (**b**) marks the position of the step edge.

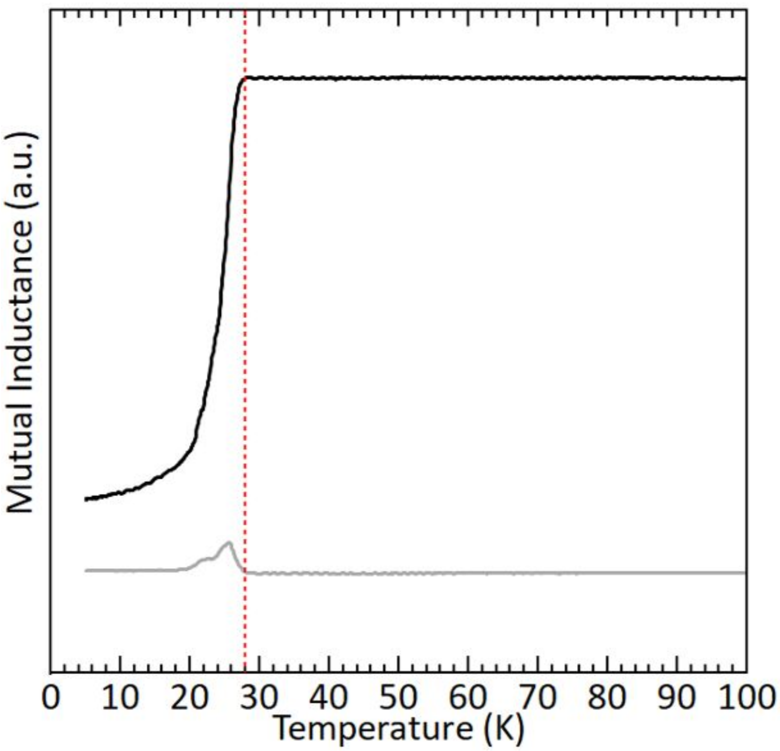

SI Figure 2: Temperature dependent mutual inductance measurement showcasing the superconducting transition at around 28K.

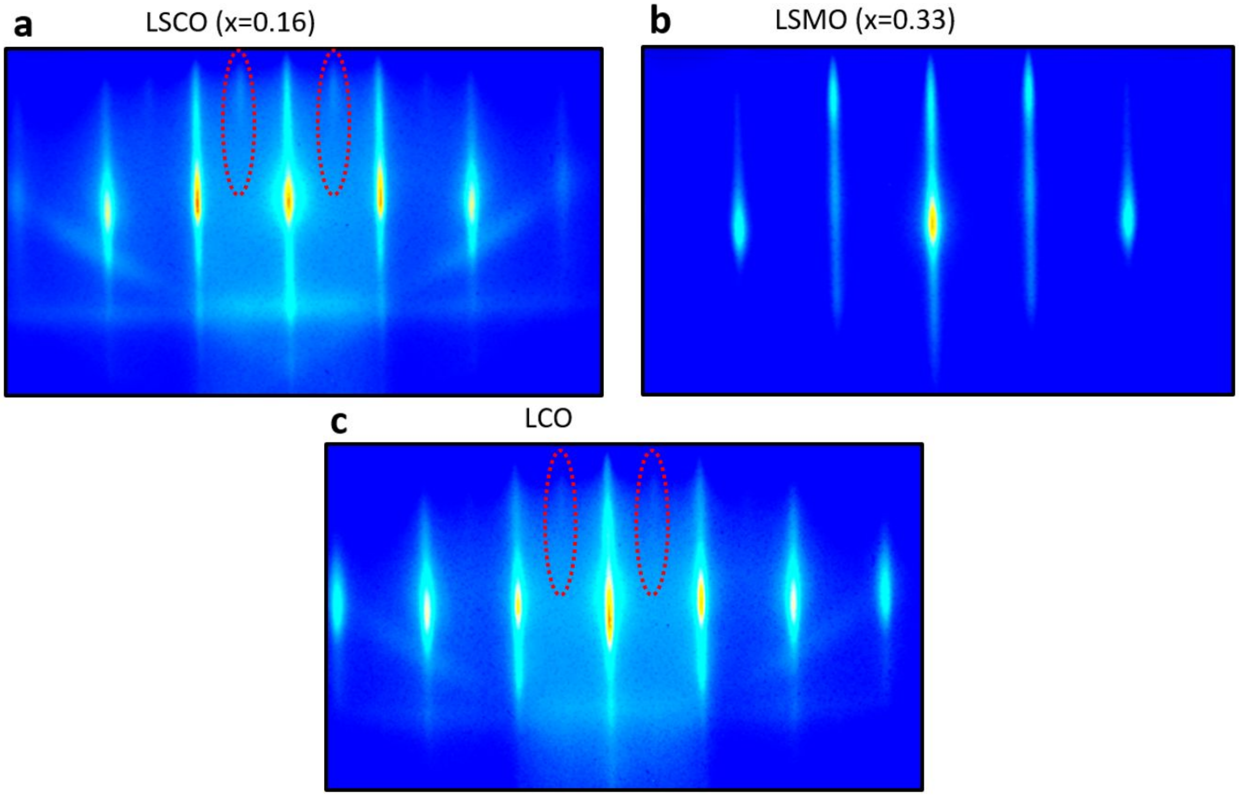

SI Figure 3: RHEED images of distinct monolayers during the growth: (**a**) 15 ML of LSCO (x=0.16), (**b**) 16 ML LSMO (x=0.33), and (**c**) 26 ML of LCO.

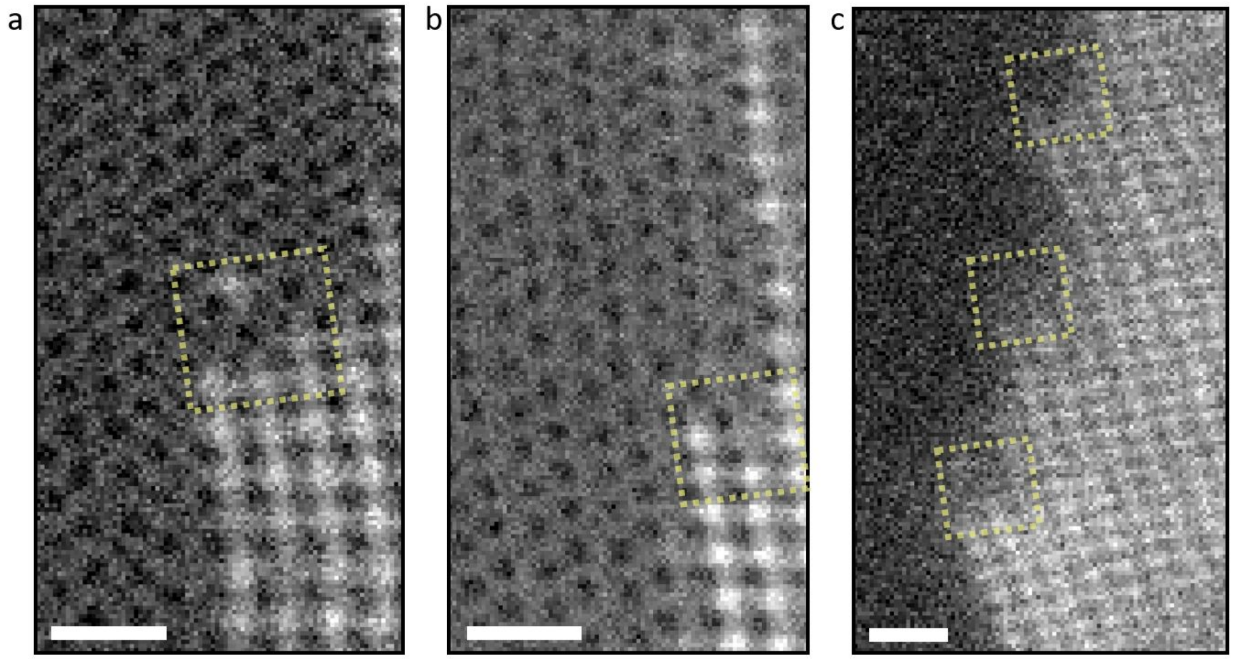

SI Figure 4: 2D mapping of the O-K pre-edge region at different positions in the sample. Different step edge (dashed yellow boxes) regions of (a) Mn atoms in $La_{1.84}Sr_{0.16}CuO_4$ matrix, (b) Cu atoms in $La_{0.66}Sr_{0.34}MnO_3$ matrix, and (c) no detectable Cu-Mn intermixing show a reduced pre-peak in the O-K edge. The energy range of the mapping was 526-529 eV, which corresponds to the green background in Figure 3b. All scale bars correspond to 1 nm.

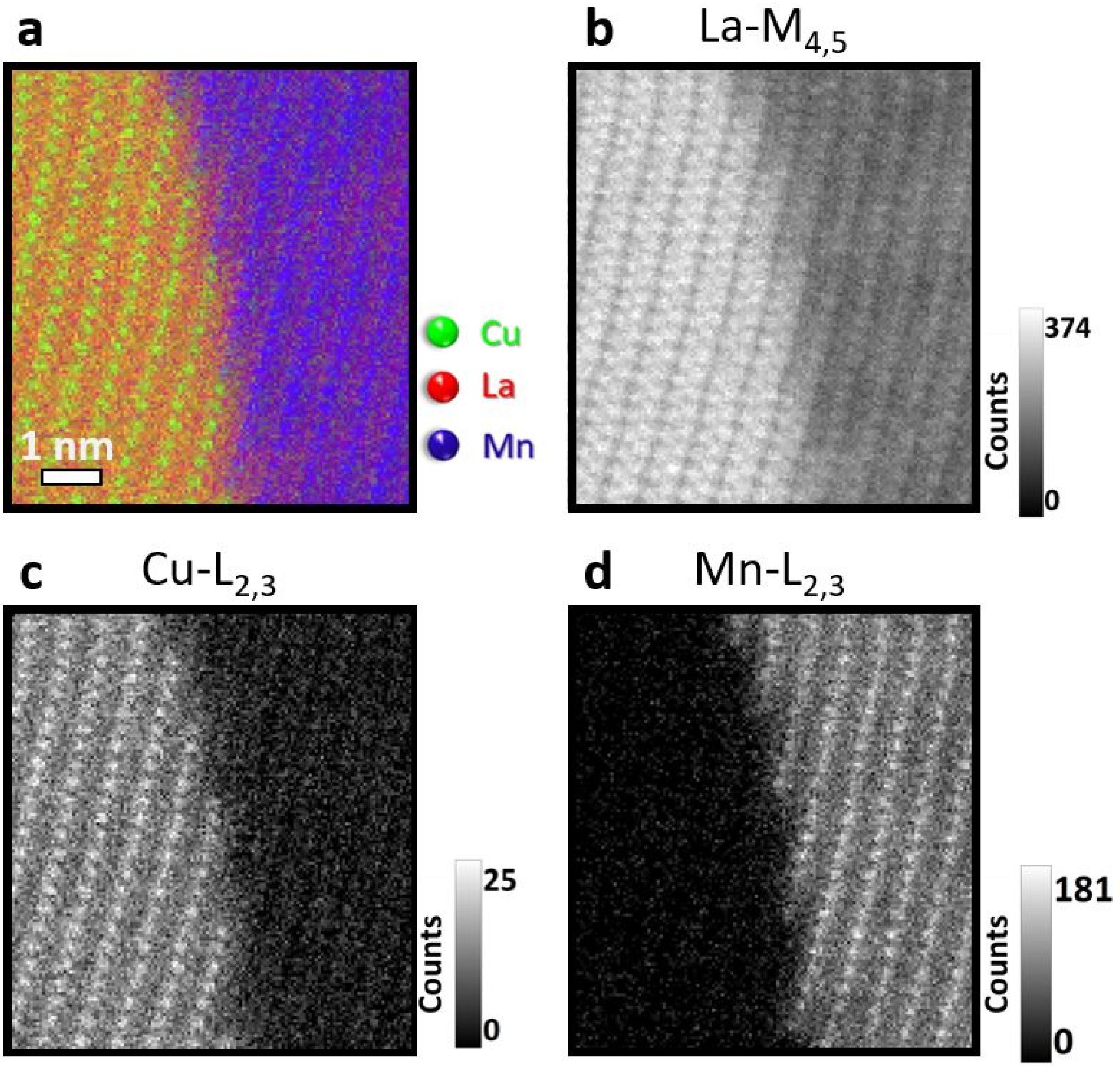

SI Figure 5: Atomically resolved EELS elemental mapping. (a) False color-coded elemental map with La in red, Cu in green, and Mn in blue. (b), (c), (d) Individual elemental maps of La, Cu, and Mn, respectively.
